# Development and validation of an EHR-based risk prediction model for geriatric patients undergoing urgent and emergency surgery

**DOI:** 10.1186/s12871-024-02880-4

**Published:** 2025-01-27

**Authors:** Edward N. Yap, Jie Huang, Joshua Chiu, Robert W. Chang, Bradley Cohn, Judith C. F. Hwang, Mary Reed

**Affiliations:** 1 Department of Anesthesia, The Permanente Medical Group, Kaiser Permanente South San Francisco, 1200 El Camino Real, South San Francisco, CA 94080 USA; 2 Department of Anesthesia and Perioperative Care, University of California San Francisco, 505 Parnassus Ave, San Francisco, CA 94143 USA; 3 Kaiser Permanente Division of Research, 2000 Broadway, Oakland, CA 94612 USA; 4 Department of Vascular Surgery, The Permanente Medical Group, 1200 El Camino Real, South San Francisco, CA 94080 USA; 5 Department of Anesthesia, The Permanente Medical Group, 3600 Broadway, Oakland, CA 94611 USA; 6 Department of Anesthesia, The Permanente Medical Group, 975 Sereno Dr, Vallejo, CA 94589 USA

**Keywords:** Risk prediction model, Geriatric surgery, Emergency surgery

## Abstract

**Background:**

Clinical determination of patients at high risk of poor surgical outcomes is complex and may be supported by clinical tools to summarize the patient’s own personalized electronic health record (EHR) history and vitals data through predictive risk models. Since prior models were not readily available for EHR-integration, our objective was to develop and validate a risk stratification tool, named the Assessment of Geriatric Emergency Surgery (AGES) score, predicting risk of 30-day major postoperative complications in geriatric patients under consideration for urgent and emergency surgery using pre-surgical existing electronic health record (EHR) data.

**Methods:**

Patients 65-years and older undergoing urgent or emergency non-cardiac surgery within 21 hospitals 2017–2021 were used to develop the model (randomly split: 80% training, 20% test). The primary outcome was a 30-day composite outcome including several postoperative major complications and mortality; secondary outcomes included common individual complications included in the primary composite outcome (sepsis, progressive renal insufficiency or renal failure, and mortality). Patients’ EHR-based clinical history, vital signs, labs, and demographics were included in logistic regression, LASSO, decision tree, Random Forest, and XGBoost models. Area under the receiver operating characteristics curve (AUCROC) was used to compare model performance.

**Results:**

Overall, 66,262 patients (median [IQR] age 78 [(70.9–84.0], female 53.9%, White race 68.5%) received urgent or emergency non-cardiac surgery (25.7% orthopedic cases, 21.9% general surgery cases). AUCROC ranged from 0.655 (Decision Tree) – 0.804 (XGBoost) for the primary composite outcome. XGBoost AUCROC was 0.823, 0.781, and 0.839 in predicting outcomes of sepsis, progressive renal insufficiency or renal failure, and mortality, respectively.

**Conclusions:**

We developed a model to accurately predict major postoperative complications in geriatric patients undergoing urgent or emergency surgery using the patient’s own existing EHR data. EHR implementation of this model could efficiently support clinicians’ surgical risk assessment and perioperative decision-making discussions in this vulnerable patient population.

**Supplementary Information:**

The online version contains supplementary material available at 10.1186/s12871-024-02880-4.

## Introduction

In the United States, over three million patients are admitted annually for an urgent or emergency surgical condition, and more than a quarter of those patients will ultimately undergo surgery [1]. Although urgent and emergency surgery represents a small percentage of all surgeries performed, they account for a disproportionate amount of postoperative morbidity and mortality [2, 3]. Furthermore, geriatric patients requiring urgent or emergency surgery are at a greater risk of poor outcomes due to a greater burden of comorbidities and higher level of frailty [4]. As the United States experiences demographic shifts toward an aging population, understanding the postoperative risk of morbidity and mortality for elderly patients being considered for urgent or emergency surgery is crucial for informing critical decisions related to the care of this growing geriatric population.

Clinical implementation of risk scores requires careful design choices to support feasibility and efficiency of use in clinical workflows. Although there are many scores that predict poor outcomes for older patients undergoing urgent and emergency surgery, to our knowledge, none are designed to be integrated within an electronic health record (EHR) to populate automatically and perform well across multiple surgical specialties [5]. For example, the clinical frailty scale has a strong predictive performance. However, its components must be collected from a patient intake form or survey and are not routinely captured consistently in an EHR prior to surgery [6–8]. Other risk scores that have been developed and used for perioperative risk assessment have further limitations. Readily used scores such as the American Society of Anesthesiologists physical status classification system have demonstrated subjectivity and have inconsistent inter-user reliability [9–11]. Other commonly used scores like the American College of Surgeons National Surgical Quality Improvement Program Surgical Risk Calculator, Physiological and Operative Severity Score for the enUmeration of Mortality and Morbidity, and the Revised Cardiac Risk Index have limitations in that their performance is unreliable in the geriatric population. They also can only predict singular outcomes such as mortality or cardiac arrest and are not specifically designed to predict outcomes in emergency surgery [12–16]. The continued improvement in the accessibility of EHR data has made analysis of large amounts of clinical data more feasible for real-time implementation, allowing for clinical decision support tools to be integrated into the EHR. This integration, absent in most common risk prediction scores, is crucial for clinicians’ ease of access and use of these tools [17].

In this study, we set out to develop and validate a risk stratification tool for geriatric patients under consideration for urgent and emergency surgery using EHR data that would be widely available at the time of perioperative decision-making. We incorporate time-sensitive clinical variables to predict the risk of major postoperative complications. This risk score, the Assessment of Geriatric Emergency Surgery (AGES) score, was developed utilizing retrospective data from a large regional integrated healthcare delivery system, Kaiser Permanente Northern California (KPNC). We hypothesize that the AGES model will reliably predict the risk of 30-day major postoperative complications in geriatric patients undergoing urgent or emergency surgery. Secondarily, we evaluate the AGES model’s performance utilizing multiple machine learning models.

## Methods

This study was approved by the Kaiser Foundation Institute’s Institutional Review Board and the requirement for written informed consent was waived. This article was prepared according to the Transparent Reporting of a multivariable prediction model for Individual Prognosis or Diagnosis guidelines [18].

### Setting

KPNC provides comprehensive hospital care to over 4.6 million patients who closely approximate the region’s diverse census demographics at 21 hospitals, two of which are Level II trauma centers [19]. The hospitals include 21 geographically dispersed medical centers across urban, suburban, and semirural areas. Surgical acuity is assigned by the surgeon at the time of the surgeon’s evaluation in the outpatient clinic or emergency room. Based on the surgeon’s clinical judgement, surgical cases are categorized as elective, urgent (surgery should be performed within 48 h), or emergency (surgery should be performed within 24 h).

### Study design and population

The retrospective cohort study included patients 65-years and older who underwent urgent or emergency non-cardiac surgery at KPNC facilities between January 1, 2017, and December 31, 2021. To examine index surgical cases, we defined our cohort to examine eligible surgeries in patients who had not had any other urgent or emergency surgery in the prior 180 days and who had been admitted within the seven days prior to surgery. For completeness of study data capture, we also required health plan membership in the year prior to surgery and in the study outcome period. To ensure the stability of risk prediction estimates and improve generalizability, we excluded surgical and procedural specialties with fewer than 100 procedures performed per year. Additionally, we excluded organ transplant surgery, trauma surgery, and surgeries not associated with a designated visit (outpatient, emergency room, or hospitalization). Because trauma surgeries have more time-sensitive workflows and the potential for unmeasurable confounding factors related to the trauma itself, we excluded trauma patients from our analysis.

### Outcomes

The primary outcome was a composite measure of 30-day postoperative major complications and mortality. Major complications included in the composite outcome were cardiac arrest, myocardial infarction, pulmonary embolism, sepsis, unplanned intubation, deep vein thrombosis, acute renal failure, and cerebrovascular accident. These complications were selected for their feasibility of extraction from the EHR and their importance to our institution’s surgical quality committees, which include a multidisciplinary team of anesthesiologists, surgeons and perioperative clinicians. We chose the primary outcome as a composite measure to provide a holistic view of overall patient risk, while individual outcomes allow clinicians and patients to discuss risks specific to their circumstance.

Individual components of the composite outcome (sepsis, renal failure, and 30-day mortality) were analyzed separately as secondary outcomes for independent assessment. We selected sepsis, renal failure, and 30-day mortality as secondary outcomes due to their higher incidence reported in the literature and their clinical relevance in our patient population. Outcomes were defined based on either individual International Classification of Diseases, Tenth Revision (ICD-10) codes and Current Procedural Terminology (CPT) codes, or with a combination of medical images, laboratory values, or medication administration (Supplement Table 1).

### Predictors

A total of 76 predictors were used in the AGES model. Patient predictors included patient age, sex, body mass index, Comorbidity Points Score, Version 2 (COPS2) score (an internally and externally validated score in predicting mortality risk based on patients’ medical diagnoses within the 12 months prior to the index surgery) and individual component of hierarchical condition category groupings used in COPS2, Laboratory Acute Physiology Score (LAPS, based on the most physiologically deranged value of 14 laboratory tests over the month before the date that surgery was requested), case class (urgent, emergency), hours from admission to surgery, admission source, surgical case service, confusion assessment method score (a screening tool for delirium), most recent lab results since admission before the index surgery (including lactate, anion gap, albumin, arterial oxygen, pH, carbon dioxide, bicarbonate, bilirubin, blood urea nitrogen, creatinine, glucose, hematocrit, sodium, troponin, white blood cell count, prealbumin, hemoglobin A1c) and most recent vital signs recorded within 24 h before the index surgery including heart rate, blood pressure, pulse oximetry, and respiratory rate [20, 21]. The AGES score builds upon a previously developed and validated score for elective surgery patients, by adding broad capture of time-sensitive clinical measures particularly relevant in urgent or emergency surgery [22].

### Model development

We built two regression-based models (logistic regression, LASSO) and three tree-based models (decision tree, random forest, and XGBoost) to predict the risk of major postoperative complications. We used a split-sample design to randomly divide the study population into training (80%) and test (20%) sets to ensure that the model can generalize well to unseen data by being trained on one data subset (training data) and evaluated on another different dataset (test data). The training set was used to build models with 10-fold cross-validation for the hyper-parameter tuning. The test set was held aside to evaluate the performance of the model which was trained on the known outcomes of 80% of the study population.

Model discrimination based on the area under the receiver operating characteristics curve (AUCROC) was used to compare model performance, along with the area under the precision-recall curve (AUCPRC) and calibration curve of the model with the highest AUCROC. To ensure fair and equitable outcomes for all populations, we also assessed AUCROC across race/ethnicity, sex, and surgery service, respectively.

Our primary goal in this study was to develop a working tool that can be used with EHR data in real-time to predict the risk of major postoperative complications, so it could be used in a clinical workflow such as the one shown in Supplement Fig. 1. Because of this, unavailable values from continuous covariates were replaced with the median value from recorded measurements. Since ordering or not ordering a specific lab test itself can also be a predictor of risk, we added a variable indicating whether the lab result was missing. We created a missing variable category for each categorical variable. Combining median imputation with missing indicators is a method that can be used when the missing data is both missing at random and missing not at random. We chose median imputation because it is a simple, efficient, and computationally feasible method for handling missing data. Combining median imputation with missing indicators is particularly useful when data is missing at random or missing not at random. This approach allows us to capture the significance of missing values while maintaining computational efficiency. Analyses were performed using SAS version 9.4 (SAS Institute), and R version 4.0.2 (The R Foundation).

## Results

A total of 95,704 patients of age 65 years or older were identified who received urgent or emergency non-cardiac surgery at KPNC from January 1, 2017, through December 31, 2021. After applying exclusion criteria, 66,262 patients remained in the study cohort (excluded cohort in Supplement Table 2).

Characteristics of the study population and selected predictor variables are presented in Tables 1 (for categorical variables) and 2 (for continuous variables). The mean age was 78 years, 53.9% were female, 68.5% were of White race/ethnicity, 6.9% were of Black race/ethnicity, 12.1% were of Hispanic race/ethnicity and 11.4% were of Asian race/ethnicity. Among the surgical services involved, 25.7% were orthopedic cases, 21.9% were general surgery cases, and 21.1% were gastroenterological procedures. Characteristics and selected predictor variables separated by the training and testing data is presented in Supplement Tables 3a (for categorical variables) and 3b (for continuous variables).

**Table 1:** Selected characteristics of patients with urgent or emergency non-cardiac surgery, 2017–2021 (categorical predictor variables), N (%)

	*Total*	*66,262*
Patient Sex	Female	35,713 (53.9)
Surgical Service	Gastroenterology	13,983 (21.1)
	General Surgery	14,478 (21.8)
	Orthopedics	17,046 (25.7)
	Head and Neck	655 (1.0)
	Interventional Radiology	6,665 (10.1)
	Neurosurgery	1,183 (1.8)
	Ophthalmology	1,089 (1.6)
	Podiatry	3,861 (5.8)
	Spine	804 (1.2)
	Thoracic	329 (0.5)
	Urology	4,538 (6.8)
	Vascular	1,631 (2.5)
Surgery Class^a^	Emergency	45,970 (69.4)
	Urgent	20,292 (30.6)
Admission Source	Outpatient	5,180 (7.8)
	Emergency Department	10,265 (15.5)
	Hospital	50,817 (76.7)
Time from admission to surgery	Within 24 Hours	37,922 (57.2)
	Within 48 Hours	15,955 (24.1)
	48 + hours	12,385 (18.7)

^a^Surgery class assigned by the surgeon: urgent (within 48 h) or emergency (within 24 h)

**Table 2:** Selected characteristics of patients with urgent or emergency non-cardiac surgery, 2017–2021 (continuous predictor variables), Median (IQR)

Total	66,262
Age	76.8 (70.9–84.0)
BMI	26 (23–31)
COPS2	39 (14–78)
LAPS	0 (0–5)
Anion Gap	8 (7–10)
Arterial Oxygen	87.0 (70.0–116.0)
Bicarbonate	23.4 (19.4–26.9)
Carbon dioxide	25.0 (21.0–29.0)
Creatinine	0.9 (0.7–1.3)
Glucose	120 (101–151)
Hematocrit	35.0 (29.8–39.3)
Hemoglobin	11.5 (9.7–13.0)
Lactate	1.4 (1.0–1.7)
pH	7.4 (7.3–7.4)
Troponin I	0.02 (0.02–0.04)
White blood cell count	9.0 (6.8–12.1)
Prealbumin	12.0 (7.9–16.9)
Heart rate	77 (68–88)
Systolic Blood Pressure	135 (120–150)
Diastolic Blood Pressure	68 (59–78)
Oxygen saturation	97 (96–99)
Respiratory rate	18 (16–19)

*Abbreviations*: *BMI* Body mass index, *COPS2* Comorbidity Points Score, Version 2, *LAPS* Laboratory Acute Physiology Score

**Fig. 1:**
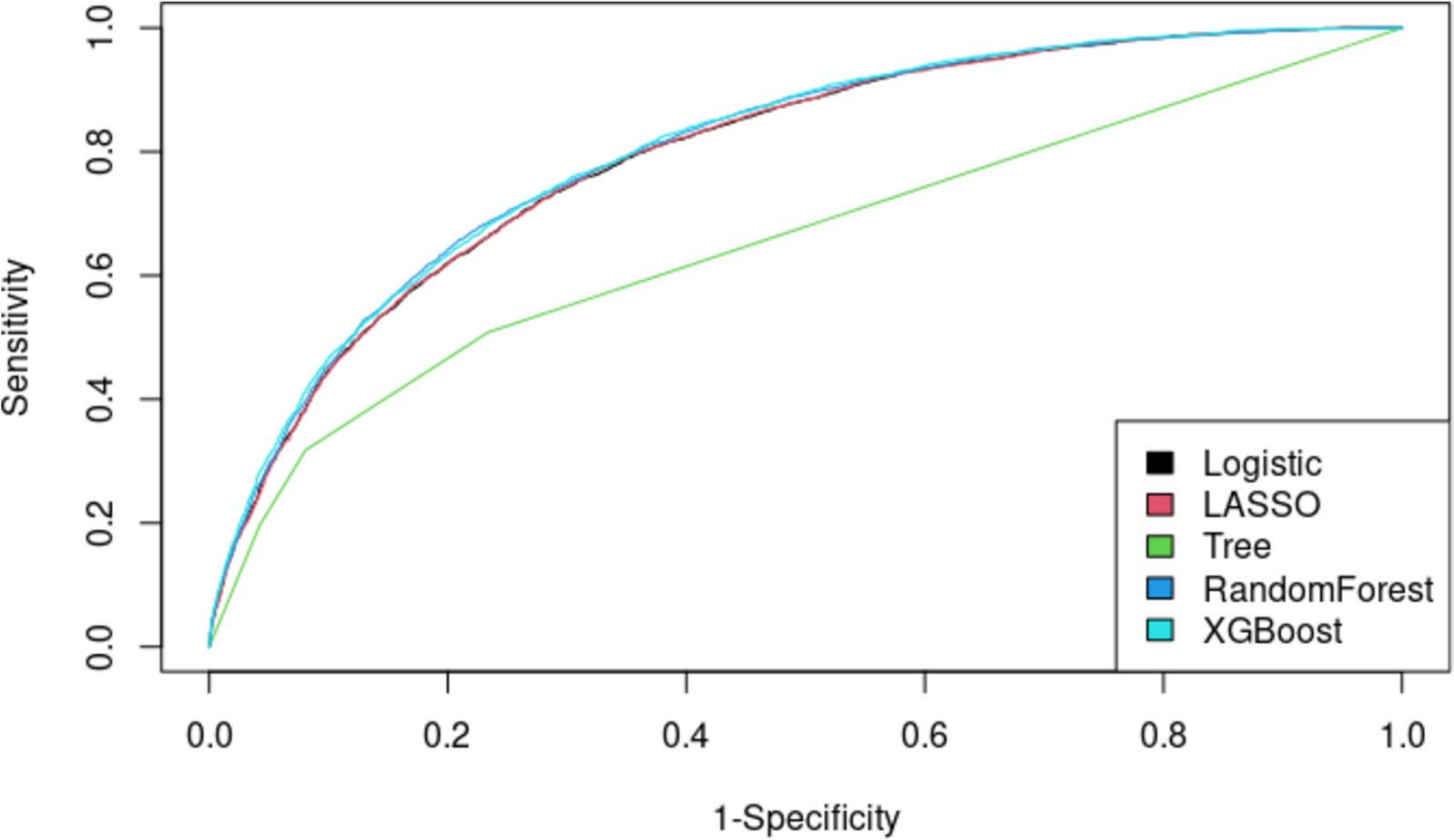
ROC Curve: composite 30-day postoperative major complications and mortality

**Fig. 2:**
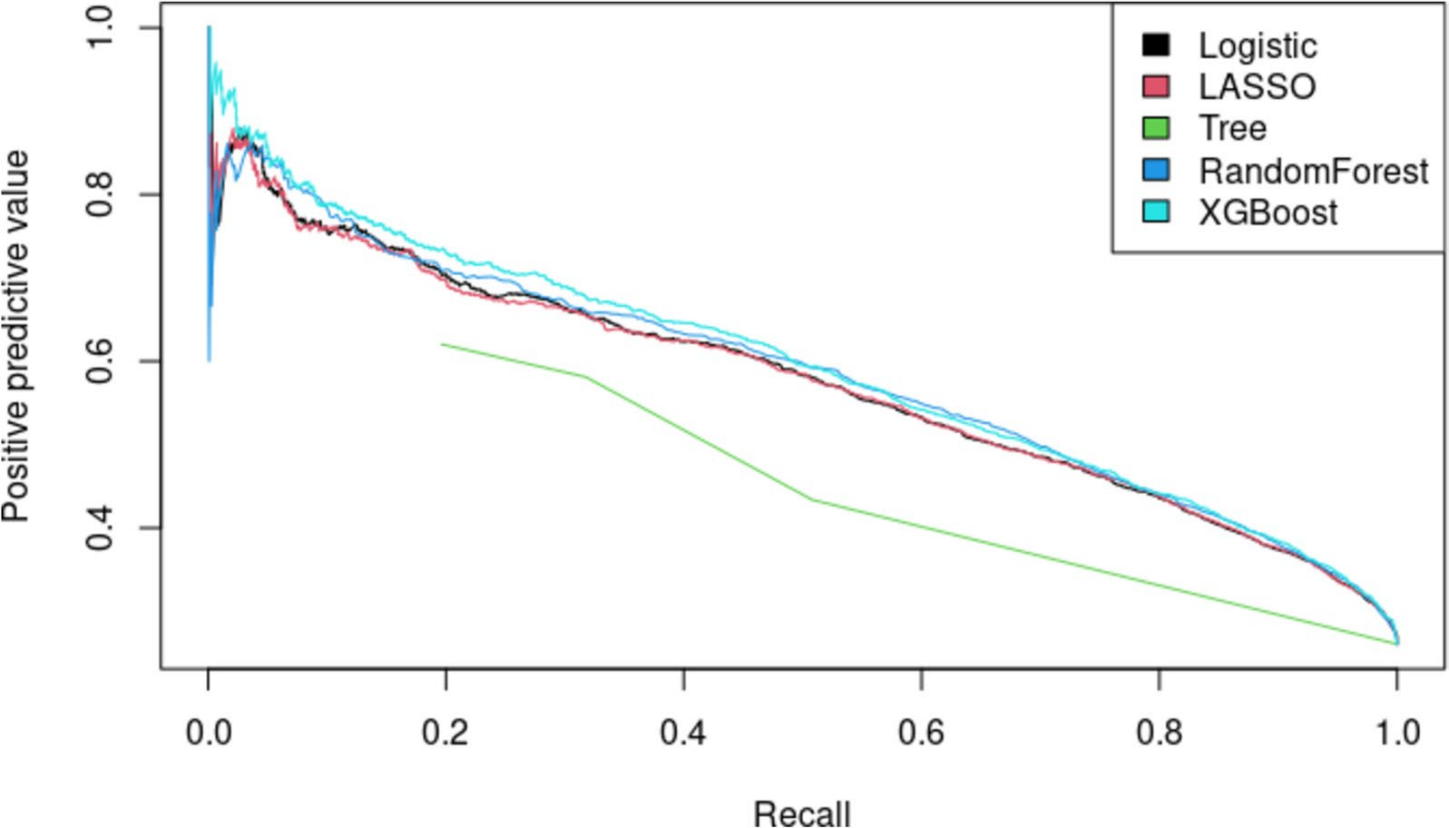
Precision Recall Curve: composite 30-day postoperative major complications and mortality

**Fig. 3:**
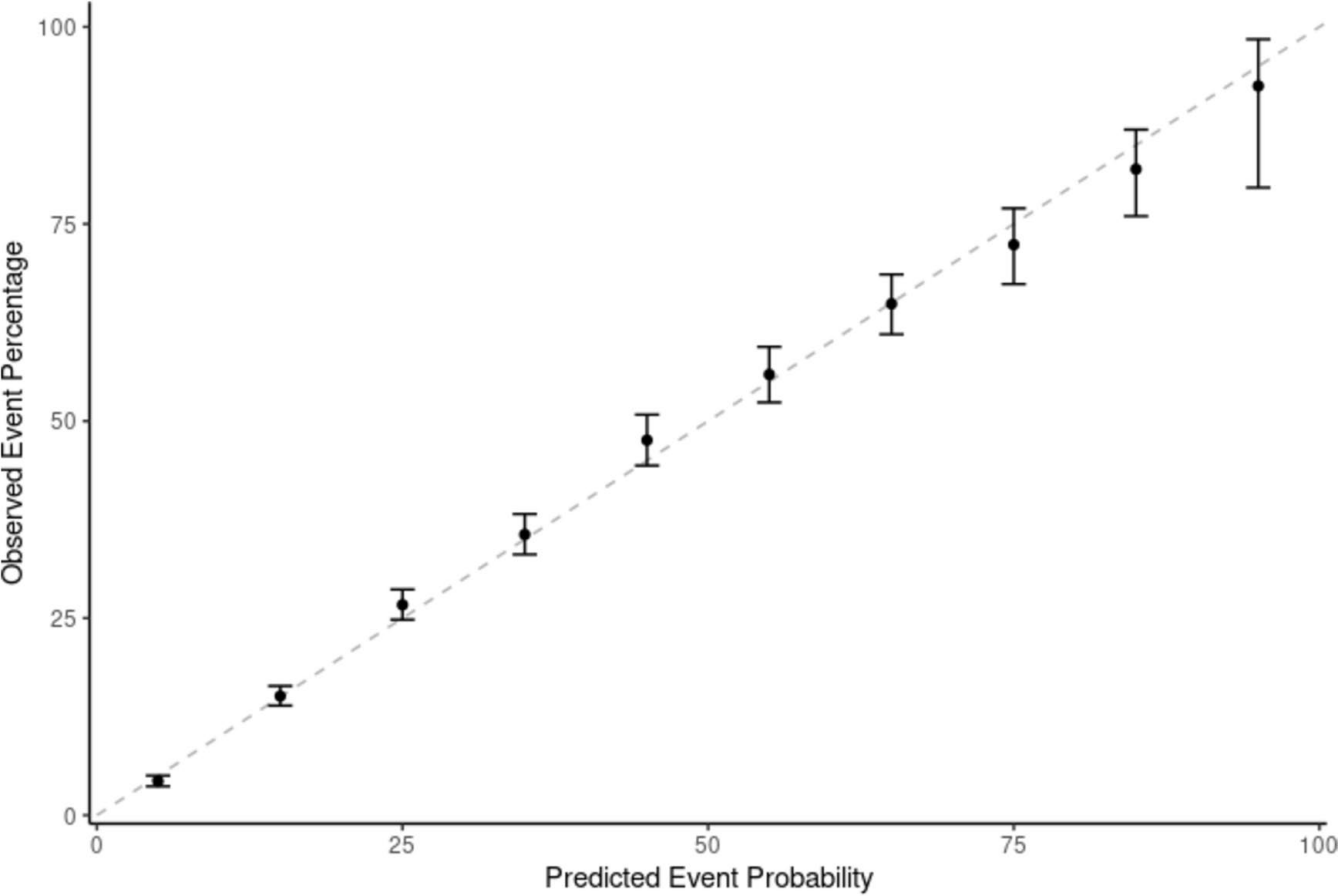
Calibration Curve: composite 30-day postoperative major complications and mortality (XGBoost)

The following results detail the performance of the tested models in predicting the 30-day risk of major postoperative complications, using the test cohort. Figure 1 presents the discrimination ability of each model, comparing how well each model predicts the primary outcome. Overall, all models demonstrated similar discrimination ability for predicting the primary outcome, except for the Decision Tree, which performed lower. The highest AUCROC was 0.804 from XGBoost, followed by 0.802 from random forest, 0.796 from logistic regression, 0.796 from LASSO, and 0.655 from Decision Tree (Table 3). Figure 2 shows the AUCPRC of each model. The highest AUCPRC was 0.593 from XGBoost, 0.583 from random forest, 0.575 from logistic regression, 0.573 from LASSO, and 0.442 from Decision Tree. Based on the model performance, we selected XGBoost as the final model used to predict the outcome. Figure 3 shows the calibration of XGBoost, demonstrating good agreement between the predicted and observed probabilities for the primary outcome. Supplement Table 4 presents crude complication rates, while Supplement Fig. 2 highlights the most influential predictor variables, enhancing interpretability of the complex XGBoost model.

**Table 3:** AUC from different models (test data) for the composite outcome

Model	AUC	95% CI	
Logistic regression	**0.796**	0.787	0.804
LASSO	**0.796**	0.788	0.804
Tree	**0.655**	0.645	0.665
Random Forest	**0.802**	0.794	0.810
XGBoost	**0.804**	0.796	0.812

Subgroup analysis of XGBoost performance in the test cohort indicated consistent predictive accuracy across race/ethnicity groups (AUCROC White: 0.802, Black: 0.794, Hispanic: 0.799, Asian: 0.814), sex (AUCROC male: 0.797, female: 0.807), or surgical services (AUCROC Orthopedics: 0.784, General surgery: 0.83, Gastroenterology: 0.775, Other: 0.797), with overlapping 95% confidence intervals.

The XGBoost model demonstrated strong predictive accuracy for individual clinical outcomes, achieving an AUCROC of 0.823 for 30-day postoperative sepsis, 0.781 for 30-day postoperative progressive renal insufficiency or acute renal failure, and 0.839 for 30-day postoperative mortality. These results indicate the XGBoost model’s potential for reliably assessing risk across multiple, clinically significant complications in geriatric patients.

## Discussion

Utilizing machine learning models and a multicenter patient cohort, we developed and validated an EHR-based risk prediction algorithm, the AGES score, for 30-day major postoperative complications and 30-day mortality in geriatric patients undergoing urgent and emergency surgery. Aside for the Decision Tree model, we achieved excellent discrimination in the prediction of a composite outcome of 30-day postoperative major complications and mortality, as well as excellent discrimination from the XGBoost model in more common individual 30-day major complications and mortality. The best performing model was XGBoost, which combines the predictions of multiple weaker models to create a stronger overall model. It does this sequentially, where each new model tries to correct the errors made by the previous ones. Like other models that have been implemented in our healthcare system, the AGES score represents an excellent model designed for the geriatric patient undergoing urgent or emergency surgery that can be integrated into the EHR [20–23]. The model performed well across different race and ethnicity groups, sex, and surgical services, which increases generalizability and addresses concerns of bias that may be seen in some machine learning models. Although the predictive performance of XGBoost was not statistically different from that of the Random Forest, logistic regression and LASSO models, we recommended XGBoost for implementation due to its best performance and its flexibility in handling complex relationships within the data and the feasibility to implement in our EHR [24]. However, we recognize that the logistic regression and LASSO are more straightforward to implement, offering advantages in terms of simplicity and interpretability. These considerations should guide future implementations based on the needs and resources of specific institutions.

Proper risk assessment is an important factor in the discussion and shared decision-making between surgeons and patients to proceed with surgery. Accurate and timely information is especially important in urgent and emergency surgery, where time may be limited due to potential clinical deterioration of the patient. Previously developed risk scores may have some utility in risk assessment; however, there are currently no risk scores designed specifically for the geriatric patient undergoing urgent and emergency surgery across multiple surgical specialties [25]. In addition, currently developed and utilized risk scores have other limitations, such as inter-user variability, poor quantitative assessment of risk, potential for underestimation of risks, or the need for clinicians to manually input data into an external non-EHR interface [25]. To address these limitations, a recent study developed and validated an automated machine-learning algorithm utilizing commonly available EHR-based patient data that accurately predicted eight major postoperative complications after surgery that could be easily implemented into an EHR for real-time clinical use [26]. Similarly, we utilized historic clinical data as well as contemporaneous, time-sensitive data (recent vital signs and laboratory results) in our predictive algorithm. We utilized multiple machine learning algorithms to model the best predictive performance. However, we focused our patient cohort on the geriatric patient undergoing urgent and emergency surgery, where a specific risk score in this patient population is lacking. A key benefit of machine learning models is their ability to account for variable interactions, making them adaptable across different surgical services.

Addressing the health needs of the aging population has been an ongoing topic of discussion that has led to policy changes and systematic initiatives to improve care for the elderly [27]. Implementation of programs that entail goal-concordant care focusing on shared decision making, preoperative workflows that help identify high-risk patients, perioperative and postoperative management focusing on improving outcomes, and the development of discharge plans to help with transitions of care have shown to be feasible and associated with improved outcomes [28–31]. A key component of such programs involve preoperative screening to assess for modifiable vulnerabilities that may influence surgical outcomes and inform decision making. This is the use-case for which we believe clinicians and patients will derive great benefit from the AGES model, which is highlighted in a case example in Supplement Fig. 1. The AGES model would give a probability of risk, which can then be stratified based on the end-user’s preference. For example, the stratification can be categorical (i.e. low, average, high, very high) or expressed as a continuous percentage that can be compared to an average. Having a real-time EHR-based risk assessment readily available and easily accessible for surgeons can help provide valuable information in the shared decision-making process of having surgery as well as in the discussion of informed consent. Being able to provide timely risk assessment in the geriatric patient undergoing urgent and emergency surgery can also potentially bolster the quality improvement efforts addressing the various unexpected postoperative complications seen in acute surgery for the elderly [32, 33].

There are several limitations of our study to be noted. The data used to develop this study’s algorithm came from a multicentered integrated health care system, which may limit generalizability of our results across different practices and settings. While our risk tool offers valuable insight, health systems must carefully evaluate the feasibility of implementing externally developed risk assessment tools within their specific settings, including consideration of EHR-developed tools. The study period encompassed the COVID-19 pandemic, which could have potentially influenced postoperative complication rates. Although our model did not have significant differences between the pre-pandemic and pandemic time frames in the test cohort (Supplement Table 5), the complexity of the pandemic’s healthcare impacts warrants further research in the post-pandemic period. The scope of this research did not include a comparison of our model to other established risk scores, which future studies should address. However, the primary outcome in our model may limit the ability to compare with other models and scoring systems. Similarly, the study’s definition of surgical class, urgent and emergency, may limit the generalizability of the model in different systems. External validation of the score in additional external clinical settings would also be needed to generalize our results to other settings. The variables used in the prediction algorithm are associated with the composite outcome studied, and therefore addressing one of these clinical variables may not necessarily change a patient’s risk profile. Similar to other models that incorporate time-sensitive variables, the timing of the risk score calculation can be limited by the availability of those variables. While the prediction model may help with risk stratification, it may not necessarily translate to improvements in surgical outcomes or quality of care. Implementation, monitoring, and recalibration of the prediction algorithm in clinical practice and evaluation of its impacts on clinical care and patient outcomes are needed.

## Conclusion

In a multicenter study of surgical outcomes in patients over age 65 undergoing urgent or emergency surgery, we developed a model using a set of EHR-derived patient history data, recent vitals, and recent lab results to predict composite serious surgical outcomes including mortality. As advancements in health technology, data infrastructure, and machine learning accelerate, it is imperative that healthcare systems evolve to seamlessly apply real-time personalized patient data to improve patient care and outcomes. Our risk prediction model provides a significant and reproducible improvement in predictive information, one which can help address the issue of a readily available, automated, multispecialty pre-operative risk assessment tool specific to the geriatric population undergoing emergency surgeries. The next steps for our research team are to assess clinical usability and acceptability by physicians caring for this vulnerable population. Further evaluation is needed to determine whether the incorporation of this risk prediction model in clinical practice can translate to improvement in patient care and outcomes.

## Supplementary Information

Supplementary Material 1.

Supplementary Material 2.

Supplementary Material 3.

Supplementary Material 4.

Supplementary Material 5.

Supplementary Material 6.

Supplementary Material 7.

## Data Availability

The datasets generated and/or analysed during the current study are not publicly available due to privacy and ethical reasons. However, they are available from the corresponding author on reasonable request.

## Abbreviations

EHR: Electronic health record
AGES: Assessment of Geriatric Emergency Surgery
KPNC: Kaiser Permanente Northern California
ICD-10: International Classification of Diseases, Tenth Revision
CPT: Current Procedural Terminology
COPS2: Comorbidity Points Score, Version 2
LAPS: Laboratory Acute Physiology Score
AUCROC: Area under the receiver operating characteristics curve
AUCPRC: Area under the precision-recall curve
