# Supplementary figures and images for: Development and validation of an EHR-based risk prediction model for geriatric patients undergoing urgent and emergency surgery
Source: BMC Anesthesiol. 2025 Jan 27;25:33. doi: 10.1186/s12871-024-02880-4 (PMC11771050; doi:10.1186/s12871-024-02880-4)

Supplement Figure 1. Case example for the use of the AGES Score

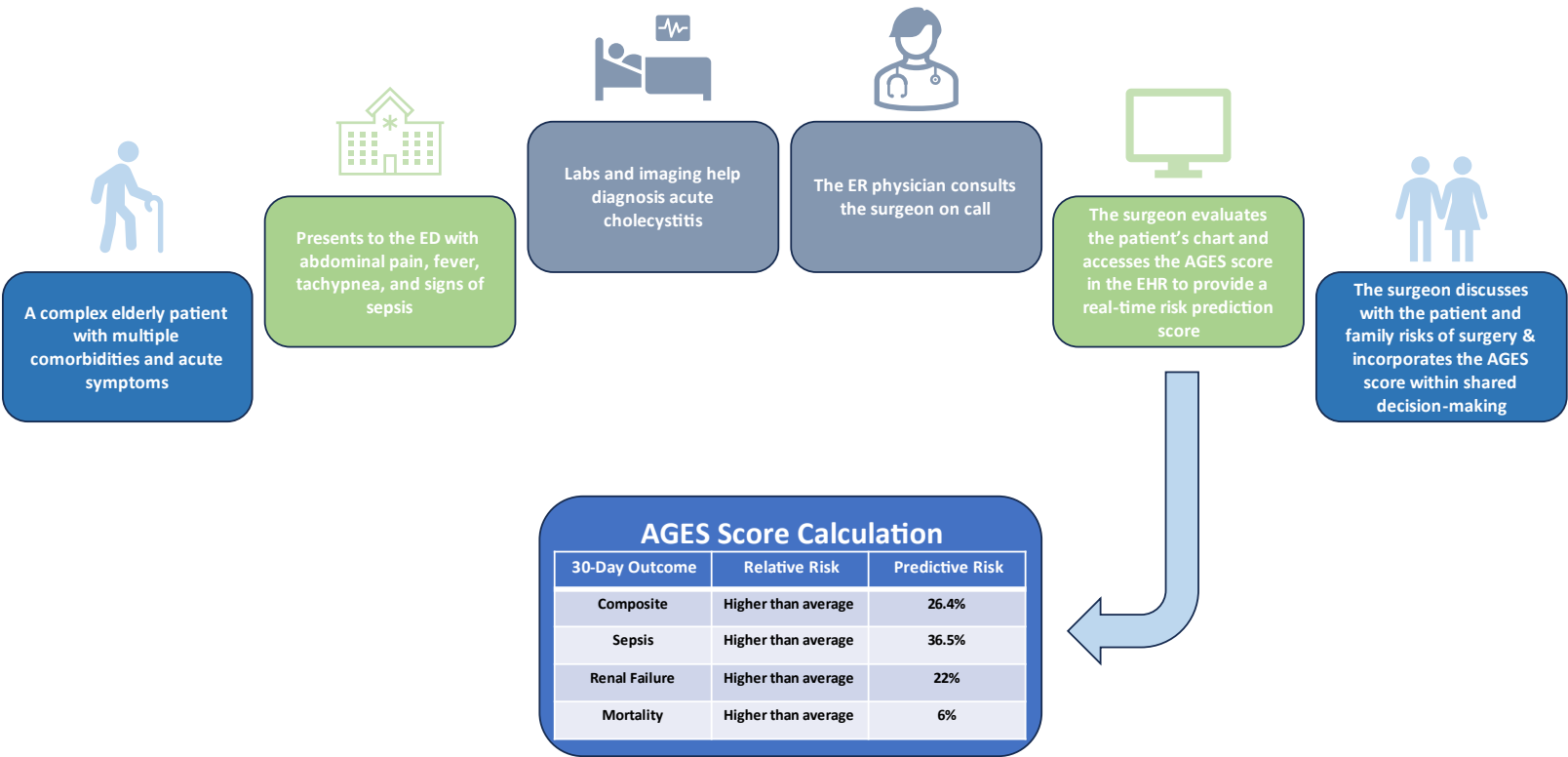

Supplement: Supplementary file 1 — Supplementary Material 1. [file 12871_2024_2880_MOESM1_ESM.pdf]

## Relative Variable Importance

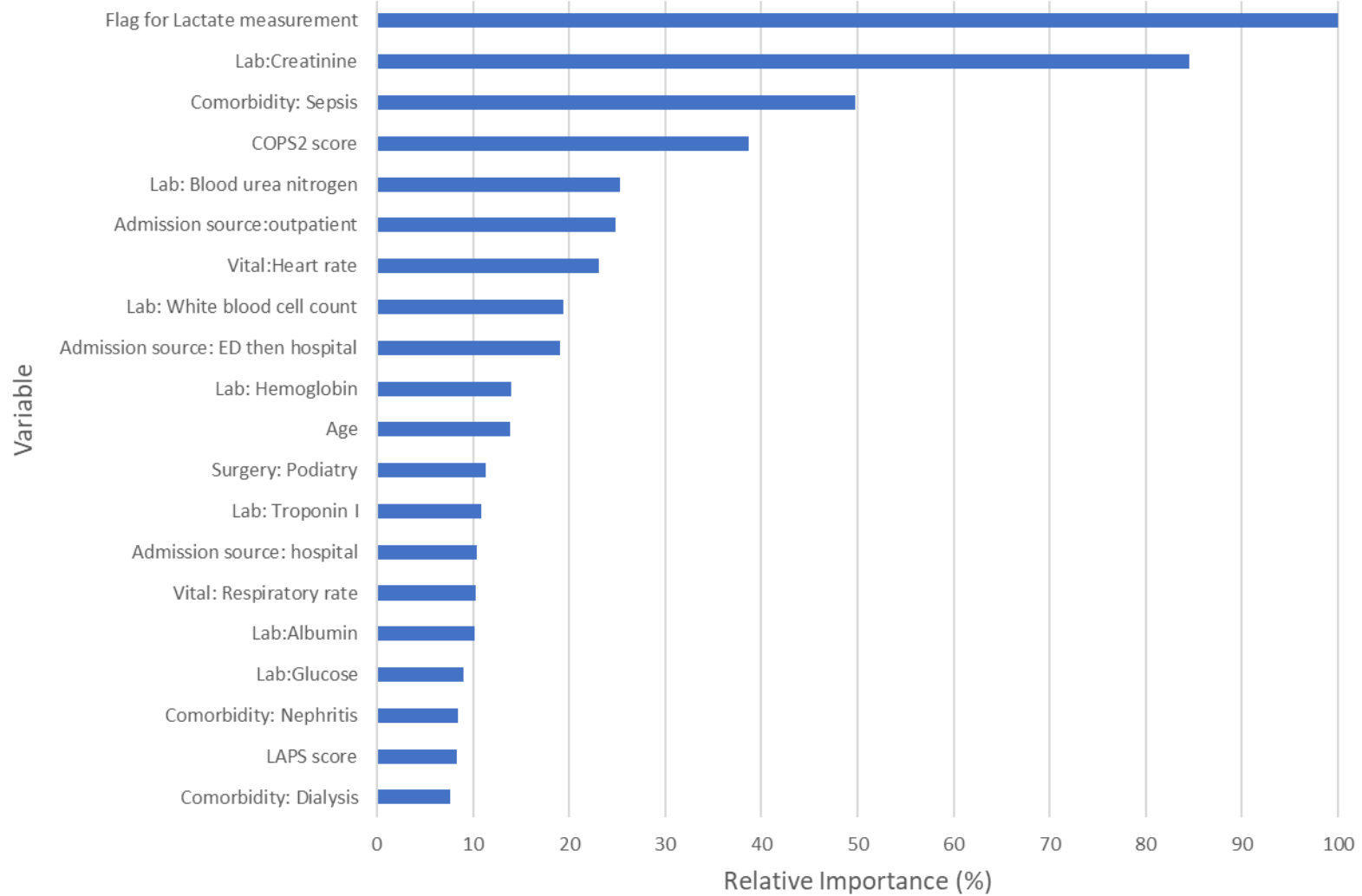

Supplement: Supplementary file 2 — Supplementary Material 2. [file 12871_2024_2880_MOESM2_ESM.pdf]
