# Supplementary material for: Development and validation of an EHR-based risk prediction model for geriatric patients undergoing urgent and emergency surgery
Source: BMC Anesthesiol. 2025 Jan 27;25:33. doi: 10.1186/s12871-024-02880-4 (PMC11771050; doi:10.1186/s12871-024-02880-4)
Supplement: Supplementary file 3 — Supplementary Material 3. [file 12871_2024_2880_MOESM3_ESM.docx]

**Supplement Table 1: Definitions for major post-operative complications used to define outcomes.**

|  | **ICD10 diagnostic codes** | **Definition** |
| --- | --- | --- |
| cardiac arrest | I46.XX, I97.711 | ICD 10 Code |
| myocardial infarction | I21.XX, I22.XX and troponin>=0.12 | ICD 10 code with new elevation in troponin >3 times the upper level of the reference range |
| pulmonary embolism | I26.XX O88.23 | ICD 10 Code with receipt of imaging either CT of the lung or VQ Scan |
| sepsis | A41.XX, T81.44, A40.XX, A42.7, A22.7, B37.7, A26.7, A54.86, B00.7, A32.7, A24.1, A20.7, A48.3, R65.20, R65.21 | ICD 10 code |
| unplanned intubation | 31500C, 224598, 234034, 246545. 31725A, 31500A, 31500D, 31500B, 207100, 31500E | Procedure code not associated with another operation within 30 days |
| deep vein thrombosis | I82.XX | ICD 10 code with receipt of an extremity ultrasound and D-Dimer > 0.49 ug/mL FEU or 500 ng/mL) or start of a new blood thinner |
| progressive renal insufficiency or acute renal failure |  | Creatinine 1.5 times baseline (24 hours prior to surgery) or rise in creatinine 0.3mg/dL. |
| cerebrovascular accident | I63.XX, I61.XX, I60.XX, I62.XX | ICD 10 code with receipt of imaging either CT or MRI of the head |
