# Supplementary material for: Development and validation of an EHR-based risk prediction model for geriatric patients undergoing urgent and emergency surgery
Source: BMC Anesthesiol. 2025 Jan 27;25:33. doi: 10.1186/s12871-024-02880-4 (PMC11771050; doi:10.1186/s12871-024-02880-4)
Supplement: Supplementary file 4 — Supplementary Material 4. [file 12871_2024_2880_MOESM4_ESM.docx]

**Supplement Table 2. Excluded cohort, patients are not exclusive to one criterion.**

| Exclusion Criteria   1. Transplant and trauma cases (n=666) 2. Case services with less than 100 procedures per year on average (n=1,642) 3. Non-KP member in the month of surgery (n=3,932) 4. With less than 9 months of membership out of 12 months prior to the surgery (n=5,736) 5. With unknown sex (n=1) 6. Surgeries not from outpatient visits, ED visits or hospitalization (n=107) 7. Having any urgent/emergent surgery in prior 180 days (n=20,943) 8. Days between admission and surgery not within 0-7 days (n=6,035)   Total exclusion N=29,442 |
| --- |
