# Supplementary material for: Development and validation of an EHR-based risk prediction model for geriatric patients undergoing urgent and emergency surgery
Source: BMC Anesthesiol. 2025 Jan 27;25:33. doi: 10.1186/s12871-024-02880-4 (PMC11771050; doi:10.1186/s12871-024-02880-4)
Supplement: Supplementary file 5 — Supplementary Material 5. [file 12871_2024_2880_MOESM5_ESM.docx]

**Supplement Table 3a. Selected characteristics of patients with urgent or emergency non-cardiac surgery in test data and train data, 2017-2021 (categorical predictor variables), N (%)**

|  |  | Test data | Train data |
| --- | --- | --- | --- |
|  | *Total* | (N=13,252) | (N=53,010) |
| Patient Sex | Female | 7,174 (54.1) | 28,539 (53.8) |
| Surgical Service | Gastroenterology | 2,797 (21.1) | 11,186 (21.1) |
|  | General Surgery | 2,894 (21.8) | 11,584 (21.9) |
|  | Orthopedics | 3,496 (26.4) | 13,550 (25.6) |
|  | Head and Neck | 160 (1.2) | 495 (0.9) |
|  | Interventional Radiology | 1,272 (9.6) | 5,393 (10.2) |
|  | Neurosurgery | 233 (1.8) | 950 (1.8) |
|  | Ophthalmology | 197 (1.5) | 892 (1.7) |
|  | Podiatry | 766 (5.8) | 3,095 (5.8) |
|  | Spine | 152 (1.1) | 652 (1.2) |
|  | Thoracic | 65 (0.5) | 264 (0.5) |
|  | Urology | 886 (6.7) | 3,652 (6.9) |
|  | Vascular | 334 (2.5) | 1,297 (2.4) |
| Surgery Class | Emergency | 9,175 (69.2) | 36,795 (69.4) |
|  | Urgent | 4,077 (30.8) | 16,215 (30.6) |
| Admission Source | Outpatient | 1,043 (7.9) | 4,137 (7.8) |
|  | Emergency Department | 2,090 (15.8) | 8,175 (15.4) |
|  | Hospital | 10,119 (76.4) | 40,698 (76.8) |
| Time from admission to surgery | Within 24 Hours | 7,619 (57.5) | 30,303 (57.2) |
|  | Within 48 Hours | 3,217 (24.3) | 12,738 (24.0) |
|  | 48+ hours | 2,416 (18.2) | 9,969 (18.8) |

**Supplement Table 3b. Selected characteristics of patients with urgent or emergency non-cardiac surgery in test data and train data, 2017-2021 (continuous predictor variables), Median (IQR)**

|  | Test data | Train data |
| --- | --- | --- |
| Total | (N=13,252) | (N=53,010) |
| Age | 76.9 (70.9-84.1) | 76.8 (70.8-84.0) |
| BMI | 26 (23-31) | 26 (23-31) |
| COPS2 | 38 (14-78) | 39 (14-78) |
| LAPS | 0 (0-5) | 0 (0-5) |
| Anion Gap | 8.0 (7.0-10.0) | 8.0 (7.0-10.0) |
| Arterial Oxygen | 86.5 (71.0-115.5) | 87.0 (70.0-117.0) |
| Bicarbonate | 23.6 (19.8-26.7) | 23.4 (19.3-26.9) |
| Carbon dioxide | 25.0 (22.0-27.0) | 25.0 (21.0-29.0) |
| Creatinine | 0.9 (0.7-1.2) | 0.9 (0.7-1.3) |
| Glucose | 120.0 (101.0-151.0) | 120.0 (101.0-151.0) |
| Hematocrit | 35.1 (29.8-39.4) | 34.9 (29.8-39.3) |
| Hemoglobin | 11.5 (9.7-13.0) | 11.5 (9.6-13.0) |
| Lactate | 1.4 (1.0-1.8) | 1.3 (1.0-1.7) |
| pH | 7.4 (7.3-7.4) | 7.4 (7.3-7.4) |
| Troponin I | 0.02 (0.02-0.04) | 0.02 (0.02-0.04) |
| White blood cell count | 9.0 (6.9-12.1) | 9.1 (6.8-12.2) |
| Prealbumin | 11.8 (7.9-16.2) | 12.1 (7.9-17.0) |
| Heart rate | 77.0 (68.0-88.0) | 77.0 (68.0-88.0) |
| Systolic Blood Pressure | 135.0 (120.0-150.0) | 135.0 (120.0-150.0) |
| Diastolic Blood Pressure | 68.0 (59.0-78.0) | 68.0 (59.0-78.0) |
| Oxygen saturation | 97.0 (96.0-99.0) | 97.0 (96.0-99.0) |
| Respiratory rate | 18.0 (16.0-19.0) | 18.0 (16.0-19.0) |
