# Supplementary material for: Development and validation of an EHR-based risk prediction model for geriatric patients undergoing urgent and emergency surgery
Source: BMC Anesthesiol. 2025 Jan 27;25:33. doi: 10.1186/s12871-024-02880-4 (PMC11771050; doi:10.1186/s12871-024-02880-4)
Supplement: Supplementary file 6 — Supplementary Material 6. [file 12871_2024_2880_MOESM6_ESM.docx]

**Supplement Table 4. Crude outcome rates in total patient cohort**

| N | 66,262 |
| --- | --- |
| Composite Outcome | 26.06% |
| Cardiac arrest | 1.03% |
| Myocardial infarction | 1.10% |
| Pulmonary embolism | 0.53% |
| Sepsis | 11.98% |
| Unplanned intubation | 0.82% |
| Deep vein thrombosis | 0.69% |
| Progressive renal insufficiency or acute renal failure | 13.35% |
| Cerebrovascular accident | 0.98% |
| Mortality | 5.68% |
