# Supplementary material for: Development and validation of an EHR-based risk prediction model for geriatric patients undergoing urgent and emergency surgery
Source: BMC Anesthesiol. 2025 Jan 27;25:33. doi: 10.1186/s12871-024-02880-4 (PMC11771050; doi:10.1186/s12871-024-02880-4)
Supplement: Supplementary file 7 — Supplementary Material 7. [file 12871_2024_2880_MOESM7_ESM.docx]

**Supplement Table 5. XGBoost AUCROC Test Cohort Divided by Pre-Pandemic (2017-2019) and Pandemic (2020-2021) Time Frames**

| Year | AUC | 95% CI | |
| --- | --- | --- | --- |
| 2017-2019 | 0.808 | 0.797 | 0.820 |
| 2020-2021 | 0.797 | 0.786 | 0.809 |
